# Supplementary data on the characterization and safety evaluation of HPPD W336, a modified 4-hydroxyphenylpyruvate dioxygenase protein, which confers herbicide tolerance, and on the compositional assessment of field grown MST-FGØ72-2 soybean expressing HPPD W336

**DOI:** 10.1016/j.dib.2018.08.035

**Published:** 2018-08-17

**Authors:** Rozemarijn Dreesen, Annabelle Capt, Regina Oberdoerfer, Isabelle Coats, Kenneth Edward Pallett

**Affiliations:** a BASF Agricultural Solutions Belgium N.V., Technologiepark-Zwijnaarde 38, B-9052 Gent, Belgium; b Bayer S.A.S., Bayer CropScience, 355 rue Dostoïevski, 06903 Sophia Antipolis, France; c BASF Agricultural Solutions Seed GmbH, Dieburger Straße 19, 64850 Schaafheim, Germany; d BASF Agricultural Solutions Seed US L.L.C., 2 T.W. Alexander Drive, Research Triangle Park, NC 27709, USA

## Abstract

Supplementary data are provided which are supportive to the research article entitled “Characterization and safety evaluation of HPPD W336, a modified 4-hydroxyphenylpyruvate dioxygenase protein, and the impact of its expression on plant metabolism in herbicide-tolerant MST-FGØ72-2 soybean” (Dreesen et al., 2018) [1]. The conducted supplementary analyses include the characterization of additional *Escherichia coli*-produced HPPD W336 protein batches used as a surrogate in HPPD W336 safety studies, the assessment of potential glycosylation and monitoring of stability in simulated intestinal fluid and during heating of the HPPD W336 protein. Furthermore, data are provided on conducted field trials and subsequent compositional analysis in MST-FGØ72-2 soybean grain of compounds related to the tyrosine degradation pathway and the metabolism of homogentisate.

**Specifications Table**

**Table:** 

Subject area	*Biochemistry, Biology*
More specific subject area	*Plant biotechnology*
Type of data	*Tables, Figures*
How data was acquired	*SDS-PAGE, immuno-blotting, mass spectrometry (Waters Q-tof Ultima; Waters Q-Tofmicro*^*TM*^*), spectrophotometry (Molecular Devices Monochromator SpectraMax*^®^*M2), Edman degradation (Applied Biosystems Procise 494 protein sequencer), liquid chromatography (Alliance HPLC, Waters)*
Data format	*Analyzed*
Experimental factors	*Protein expressed in E. coli and purified by means of classical chromatography. Grain sampling of field grown soybean plants*
Experimental features	*Physico-chemical and functional analysis of bacterially-produced protein*
	*Field trial and compositional analysis of soybean lines*
Data source location	*Field trials 2009: Perry (Dallas County, IA, USA); Adel (Dallas County, IA, USA); Winterset (Madison county, IA, USA), Fithian (Vermillion county, IL, USA), Sharpsville (Tipton County, IN, USA) and Mediapolis(Des Moines County, IA, USA)*
	*Field trials 2013: York (York county, NE, USA), Richland (Jefferson County, IA, USA), Leonard (Shelby county, MO, USA) Fisk (Butler county, MO, USA), Kirksville (Adair County, MO, USA), Carlye (Clinton County, IL, USA), Stewardson (Shelby county, IL, USA) and Ladoga (Montgomery county, IN, USA)*
Data accessibility	*The data are available with this article*
Related research article	*[1]**R. Dreesen, A. Capt, R. Oberdoerfer, I. Coats, K.E. Pallet, Characterization and safety evaluation of HPPD W336, a modified 4-hydroxyphenylpyruvate dioxygenase protein, and the impact of its expression in herbicide-tolerant MST-FGØ72-2 soybean on plant metabolism, Reg. Toxicol. and Pharmacol., 97, 2018, 170–185.*

**Value of the data**

• Use of bacterially-produced proteins as fit-for-use surrogates for low-expressing plant proteins.
• Description of tools commonly used in a regulatory safety assessment of a genetically modified trait to provide transparency in the regulatory process applied by many countries.
• Evaluating the role of a ubiquitous enzyme such as HPPD (4-hydroxyphenylpyruvate dioxygenase) through over-expression may lead to a deeper understanding of a key metabolic pathway in plants and the role the enzyme plays in this pathway.

## 1 Data

The provided data are supplementary to the data described in [1].

**Fig. 1:**
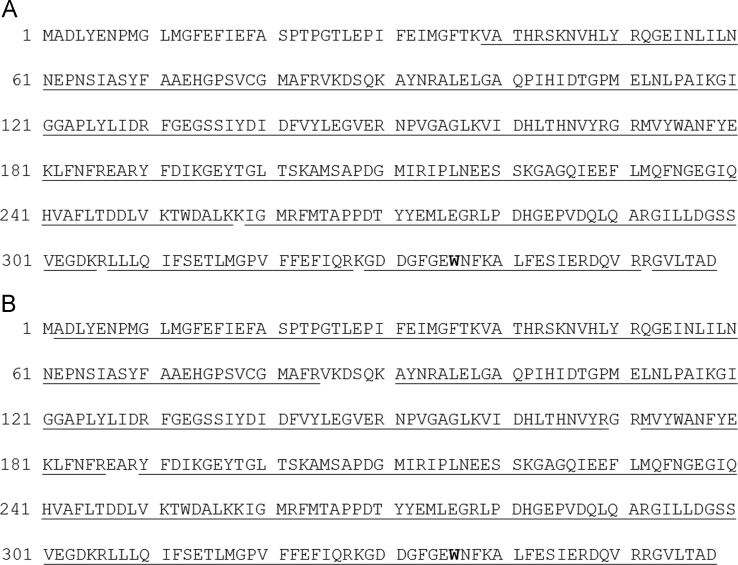
MALDI-TOF coverage of *E. coli*-produced batches HPPD W336-2 and -3. MALDI-TOF coverage results of the theoretical HPPD W336 sequence by tryptic peptides generated for *E. coli*-produced protein batch HPPD W336-2 and HPPD W336-3 are shown in panel A (88.0%) and panel B (96.1%), respectively. Underlined regions correspond to tryptic peptides that were identified using MALDI-TOF MS.

**Fig. 2:**
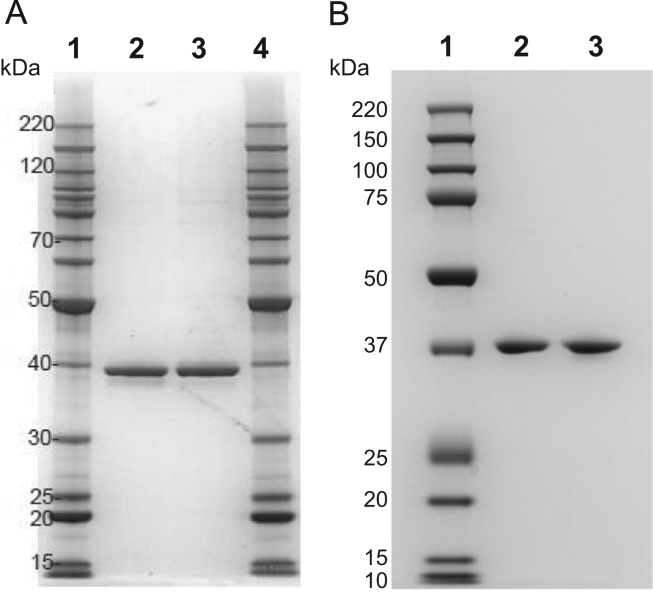
Comparative assessment of apparent molecular masses of *E. coli* produced protein batches HPPD W336-2 and -3 in relation to HPPD W336-1 by SDS-PAGE. Aliquots of *E. coli*-produced HPPD W336 protein batches were separated by SDS-PAGE using a 10% Bis-Tris gel and MOPS SDS running buffer. The gel was Coomassie-stained. Panel A – Comparative assessment of HPPD W336-1 and HPPD W336-2. Lane 1 and 4: Molecular mass marker; Lane 2: 1 µg of *E.coli*-produced protein batch HPPD W336-1; Lane 3: 1 µg of *E.coli*-produced protein batch HPPD W336-2. Panel B – Comparative assessment of HPPD W336-1 and HPPD W336-3. Lane 1: Molecular mass marker; Lane 2: 1 µg of *E.coli*-produced protein batch HPPD W336-3; Lane 3: 1 µg of *E.coli*-produced protein batch HPPD W336-1.

**Fig. 3:**
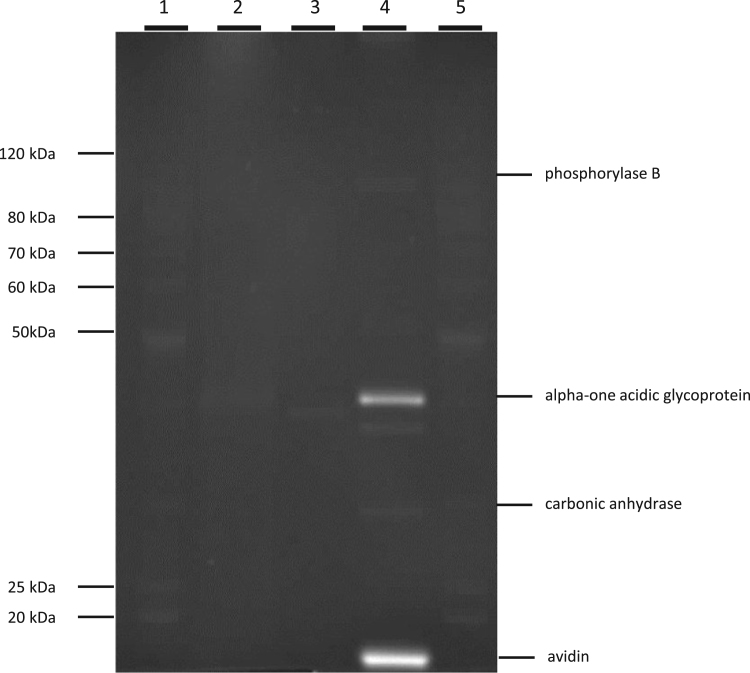
Glycosylation analysis of *E. coli*-produced HPPD W336 and MST-FGØ72-2 soybean-purified HPPD W336. N-glycosylated proteins for avidin and alpha acidic glycoprotein (positive controls) in lane 4 are seen as brightly fluorescent bands under UV lighting. Bands from the two HPPD W336 protein batches (lanes 2 and 3) and the negative controls (phosphorylase B and carbonic anhydrase; lane 4) are not brightly fluorescent, hence not glycosylated. Lanes 1 and 5: molecular mass marker. Lane 2: ~ 400 ng of MST-FGØ72-2 soybean-purified HPPD W336; Lane 3: ~ 400 ng of *E. coli*-produced HPPD W336 protein batch HPPD W336-1. Lane 4: BTS-4 Glycosylated Protein Marker.

Physico-chemical and functional characteristics of *Escherichia coli*-produced HPPD W336 batches HPPD W336-2 and HPPD W336-3 were determined (Fig. 1, Fig. 2; Table 1). Fig. 3 shows the glycostaining results for bacterially-produced HPPD W336 in relation to MST-FGØ72-2 soybean-purified HPPD W336.

**Table 1:** Summary of structural and functional characteristics of *E. coli*-produced HPPD W336 batches HPPD W336-2 and -3 used for safety assessment of the HPPD W336protein [1].

**HPPD protein batch No.**	**Characteristic and applied method**					
	**Identity**		**Protein purity (% HPPD W336/total protein)**	**Apparent molecular mass**	**Immuno-reactivity**	**Activity**
	**MALDI-TOF MS (% coverage of theoretical HPPD W336 sequence)**	**Edman degradation**^a^	**Densitometry of Coomassie-stained SDS-PAGE**	**SDS-PAGE**	**Western blot**	**Qualitative activity assay**
HPPD W336-2	88.0	ADLYE	96	40.3 kDa	Immuno-reactive band of approx. 40.3 kDa	Active
HPPD W336-3	96.1	ADLYENPMGL	99			

a The N-terminal sequence obtained for all bacterially-produced HPPD W336 batches lacks the predicted N-terminal methionine, which is often observed in proteins expressed in prokaryotic and eukaryotic organisms [2].

**Fig. 4:**
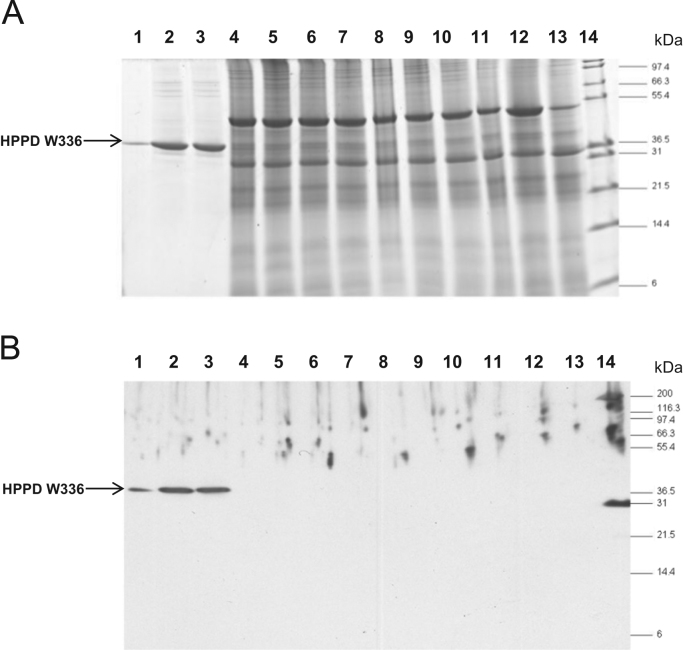
Simulated Intestinal Fluid analysis of HPPD W336. Panel A – Coomassie-stained SDS-PAGE. Panel B: western blot. Lane 1: 10% loading control with HPPD W336 (~ 100 pg), time 0, no pancreatin; Lane 2: positive control being HPPD W336, time 0, no pancreatin (~ 1 ng); Lane 3: positive control being HPPD W336, time 60 min., no pancreatin (~ 1 ng); Lane 4: HPPD W336, time 0 (~ 1 ng); Lane 5: HPPD W336, time 0.5 min.; Lane 6: HPPD W336, time 2 min.; Lane 7: HPPD W336, time 5 min.; Lane 8: HPPD W336, time 10 min.; Lane 9: HPPD W336, time 20 min.; Lane 10: HPPD W336, time 30 min.; Lane 11: HPPD W336, time 60 min.; Lane 12: negative control being SIF solution, no protein, time 0; Lane 13: negative control being SIF solution, no protein, time 60 min.; Lane 14: molecular mass marker.

**Fig. 5:**
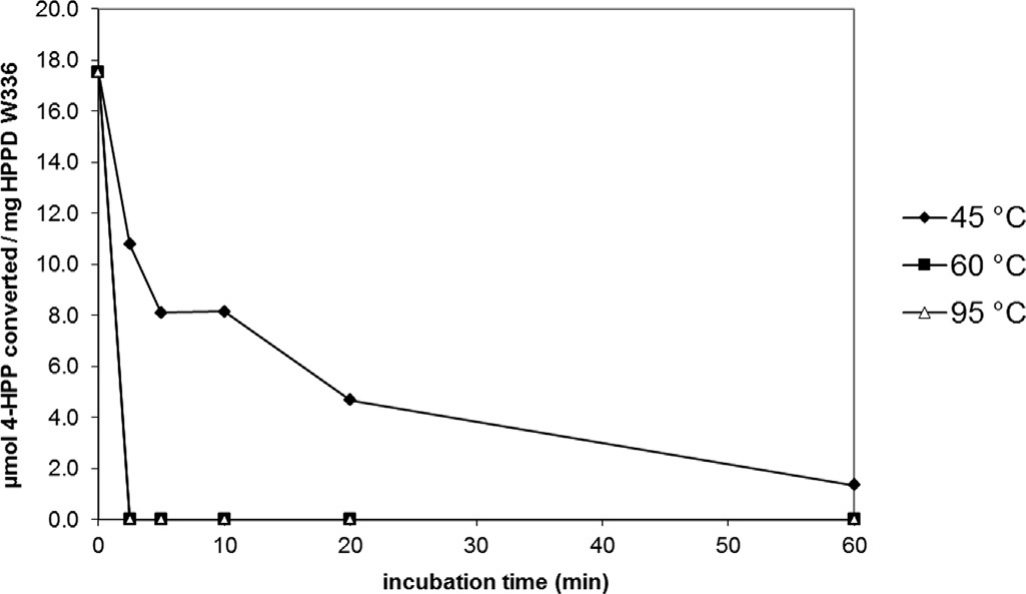
Temperature stability of the HPPD W336 protein. HPPD specific activity of assayed protein samples is expressed in µmol 4-HPP converted/mg HPPD W336 and plotted over time of heat treatment (varying from 2.5 to 60 min). Three different heat treatments were applied (45 °C, 60 °C and 95 °C).

The stability of HPPD W336 was tested under different conditions comparable to digestion and heat treatment of food. Stability analysis of in human simulated intestinal fluid containing pancreatin is shown in Fig. 4, The remaining HPPD functionality of *E. coli*-produced HPPD W336 samples which received different heat treatments is shown in Fig. 5.

Amino acid levels of herbicide treated and untreated MST-FGØ72-2 soybean grain as well as grain of conventional soybean varieties were determined, which are summarized in Table 2. Details on locations and applied field trial conditions in relation to described in [1] are shown in Table 3.

**Table 2:** Amino acid contents of MST-FGØ72-2 soybean grain in relation to herbicidal treatment and conventional soybean varieties.

**Component (mg/g dw)**	**Conventional counterpart**^a^**Not treated with Herbicides - Entry A**				**MST-FGØ72-2 soybean Not treated with Test Herbicides - Entry B**					**MST-FGØ72-2 soybean Treated with Test Herbicides**^b^**- Entry C**				**Range commercial Soybean varieties**^c^	**ILSI Version 5.0 (2014)**	**Comparison *t*-test Entry A vs B**^d^	**Comparison *t*-test Entry A vs C**^d^
	**Mean ± St Dev**				**Mean ± St Dev**					**Mean ± St Dev**				**(min-max)**	**(min-max)**	***p*-value**	***p*-value**
Tyrosine	13.4 ± 0.7				13.5 ± 0.8					13.4 ± 0.6				12.0–15.7	7.4–23.2	0.333	0.941
Phenylalanine	19.9 ± 1.3				20.1 ± 1.5					20.0 ± 1.2				17.6–24.1	15.0–25.8	0.209	0.631
Tryptophan	5.90 ± 0.38				5.88 ± 0.31					5.84 ± 0.29				4.90–7.46	2.54–7.31	0.677	0.337
Alanine	17.3 ± 0.9				17.5 ± 1.0					17.5 ± 0.8				15.5–20.9	13.1–21.5	0.121	0.218
Arginine	27.4 ± 2.2				27.9 ± 2.7					27.7 ± 2.2				24.0–36.1	19.5–39.3	0.179	0.464
Aspartic acid	45.3 ± 3.0				45.9 ± 3.3					45.5 ± 2.6				40.6–60.7	32.9–63.2	0.227	0.651
Cystine	5.59 ± 0.25				5.63 ± 0.24					5.63 ± 0.35				5.34–6.88	3.2–9.3	0.599	0.581
Glutamic acid	71.4 ± 5.2				72.0 ± 5.5					71.7 ± 4.8				62.1–95.0	43.5–102.0	0.413	0.702
Glycine	17.4 ± 1.0				17.6 ± 1.1					17.5 ± 0.8				15.4–21.6	13.0–25.5	0.189	0.537
Histidine	10.55 ± 0.61				10.74 ± 0.72					10.64 ± 0.56				9.41–13.54	2.0–15.9	0.102	0.396
Isoleucine	17.8 ± 1.1				18.2 ± 1.3					18.0 ± 1.1				15.4–21.7	13.2–24.8	0.130	0.447
Leucine	29.9 ± 1.8				30.3 ± 2.1					30.1 ± 1.7				26.7–36.0	22.6–38.9	0.145	0.409
Lysine	27.9 ± 1.6				28.4 ± 2.0					28.6 ± 1.4				25.7–35.0	17.9–39.4	0.117	0.022^e^
Methionine	5.46 ± 0.28				5.60 ± 0.31					5.50 ± 0.29				4.91–6.72	2.9–11.5	0.085	0.640
Proline	19.8 ± 1.3				19.9 ± 1.4					19.8 ± 1.2				17.2–29.5	14.4–26.3	0.563	0.683
Serine	20.4 ± 1.5				20.5 ± 1.3					20.4 ± 1.2				17.8–25.7	8.6–26.9	0.952	0.930
Threonine	16.0 ± 0.9				16.1 ± 1.0					16.0 ± 0.8				13.9–19.8	11.0–21.8	0.180	0.742
Valine	18.4 ± 1.2				18.8 ± 1.3					18.6 ± 1.3				15.3–22.5	14.2–26.6	0.162	0.496

St Dev = standard deviation.

a Non-GM conventional counterpart in genetic background, soybean line MST39.

b Foliar application of isoxaflutole (70 g ai/hectare) + glyphosate (Roundup PowerMAX; 1032 g ai/hectare) at 4–5 leaf stage.

c Either Stine^®^ 35E23, Stine^®^ 29E22 and Stine^®^ 35E32 or Stine^®^ 33E22, Stine^®^ 31E22 and Stine^®^ 30E32.

d *t*-test *p*-value: pairwise comparison to the non-GM conventional counterpart (entry A).

e A *p*-value < 0.05 was observed, which indicates a significant difference between means.

**Table 3:** Production conditions of soybean grain for composition analysis (2013) in relation to homogentisate analysis (2009, [1]).

**Field trial experiment 2013**	**Field trial experiment 2009**
(Grain production for compositional analysis of amino acids (this work) and tocochromanols [1])	(Grain production for homogentisate analysis [1])
8 sites in USA	6 sites in USA
MST-FGØ72-2 soybean and non-GM conventional counterpart in genetic background, soybean line “MST39”	MST-FGØ72-2 soybean and non-GM conventional counterpart in genetic background, soybean line “Jack”
Maturity Group 2 - 4	Maturity Group 2–3
Inclusion of 6 commercial reference varieties:	Inclusion of 3 commercial reference varieties:
Stine^®^ 35E23, Stine^®^ 29E22, Stine^®^ 35E32, Stine^®^ 33E22, Stine^®^ 31E22 and Stine^®^ 30E32	Stine^®^ 2500-2, Stine^®^ 3300-2 and Stine^®^ 3308-2
RCBD design; 4-fold replication;	RCBD design; 3-fold replication;
Reference varieties included in RCBD design; 3 out of 6 reference varieties included at each trial site	Reference varieties not included in RCBD design, but planted in separate plots alongside the RCBD trials.
Application of the test herbicides on a subset of MST-FGØ72-2 soybean plots:	Application of the test herbicides on a subset of MST-FGØ72-2 soybean plots:
One IFT application (70 g ai/hectare) pre-emergence (BBCH 01 to BBCH 08); One glyphosate application (Roundup	One IFT (70 g ai/hectare) and one Glyphosate application (Roundup Original Max; 1060 g ai/hectare)
PowerMAX; 1032 g ai/hectare) at BBCH 14 to BBCH 15;	both applied at BBCH 14 to BBCH 15;
Adjuvant Ammonium sulfate added (1426 g ai/hectare)	Adjuvant Ammonium sulfate added (1426 or 2850 g ai/hectare)
Sample shipment at ambient or frozen conditions to composition lab.	Sample shipment at ambient conditions to Bayer CropScience LP, transferred to frozen storage and shipped frozen on dry ice to composition lab.

ai: active ingredient; BBCH: scale for coding the phenological growth stages of plants; RCBD: randomized complete block design.

## 2 Experimental design, materials and methods

Physicochemical and functional characterization of HPPD W336 was performed as described in [1].

Glyco-staining analysis was performed by staining of an SDS-PAGE gel using the GlycoProfile™ III Fluorescent Glycoprotein Detection Kit (Sigma, Sint-Louis, MO).

Simulated Intestinal Fluid analysis was performed in compliance to published guidelines [3]. *E. coli-*produced batch HPPD W336-2 samples (2.5 mg/mL) were added to a solution containing 6.8 g/l KH_2_PO_4_, pH 7.5 and 1% w/v pancreatin and incubated at 37 °C. Sampling occurred at 0.5, 2, 5, 10, 20, 30 and 60 min. Positive and negative controls were included, in addition to a 10% loading control. Samples were analyzed by SDS-PAGE and western blot as described in [1].

HPPD functionality of heated samples was assessed by a semi-quantitative colorimetric activity assay according to [1], using 4-hydroxyphenylpyruvate as a standard. Different aliquots of *E. coli*-produced batch HPPD W336-1 were incubated at 45, 60 and 95 °C for 2.5, 5, 10, 20 and 60 min.

Amino acid content of soybean grain was determined in grain sampled from two field trials ([1], Table 3) The material was harvested at maturity. Samples were prepared by grinding 0.5 g of frozen grain with liquid nitrogen. Further analysis was done according to AOAC Official Methods [4], [5], [6]. Data were subjected to analysis of variance as described in [1].

## References

[1] Dreesen R., Capt A., Oberdoerfer R., Coats I., Pallet K.E. (2018). Characterization and safety evaluation of HPPD W336, a modified 4-hydroxyphenylpyruvate dioxygenase protein, and the impact of its expression in herbicide-tolerant MST-FGØ72-2 soybean on plant metabolism. Reg. Toxicol. Pharmacol..

[2] Bradshaw R.A., Brickey W.W., Walker K.W. (1998). N-terminal processing: the methionine amino peptidase and N-acetyl transferase families. Trends Biochem. Sci..

[3] Thomas K., Aalbers M., Bannon G.A., Bartels M., Dearman R.J., Esdaile D.J., Fu T.J., Glatt C.M., Hadfield N., Hatzos C., Hefle S.L., Heylings J.R., Goodman R.E., Henry B., Herouet C., Holsapple M., Ladics G.S., Landry T.D., MacIntosh S.C., Rice E.A., Privalle L.S., Steiner H.Y., Teshima R., Van Ree R., Woolhiser M., Zawodny J. (2004). A multi-laboratory evaluation of a common in vitro pepsin digestion assay protocol used in assessing the safety of novel proteins. Regul. Toxicol. Pharmacol..

[4] AOAC Authors (2006). Official Methods of Analysis Amino Acids Analysis Cysteine & Methionine (CM) - Item 73, Reference Data: Method 982.30 E(b); NFNAP; NITR.

[5] AOAC Authors (2006). Official Methods of Analysis Amino Acids Analysis Feed (OPA Post Column) - Item 81, Reference Data: Method 994.12 (4.1.11); NFNAP; NITR.

[6] AOAC Authors (2006). Official Methods of Analysis Amino Acids Analysis Tryptophan (T) Food and Feed Ingredients - Item 75, Reference data: Method 988.15; NFNAP; NITR; TRP.

